# Distal foot segment joint coupling patterns during walking gait

**DOI:** 10.1186/1757-1146-7-S1-A94

**Published:** 2014-04-08

**Authors:** Stephen C Cobb, Robin L Bauer, Mukta N Joshi

**Affiliations:** 1 Department of Kinesiology, University of Wisconsin-Milwaukee, Milwaukee, WI 53201, USA; 2 Robin Bauer was a graduate student in the MS Kinesiology program at the University of Wisconsin-Milwaukee at the time of the study

## Background

Several surface based multi-segment foot models have been developed to investigate distal foot function during gait [1,2]. However, the majority of the models have not defined medial and lateral forefoot or midfoot segments. In addition very little, is currently known regarding the coupling of the distal foot segments [3,4]. The purpose of the current study, therefore, was to utilize a six foot segment model that includes both medial and lateral forefoot and midfoot segments to quantify the coupling between the distal foot segments during walking gait.

## Methods

Ten participants (5 m, 5 f; mean age 22.7 ± 3.3 y) participated in the study. A 10 camera Motion Analysis system was used to capture three-dimensional positions of marker clusters located on the leg and six foot segments of interest (calcaneus, navicular, 1^st^ and 2^nd^ metatarsals, hallux, 4^th^ and 5^th^ metatarsals, cuboid). Following completion of 10 successful walking trials, joint coupling between adjacent segments of interest were investigated using vector coding. Repeated measures ANOVAs with one within-subject variable (stance subphase) were performed for each joint couple of interest to investigate joint coupling between stance subphases. Dependent t-tests were performed to investigate significant omnibus F ratios (α = 0.05).

## Results

Significant joint coupling differences were revealed between stance subphases for the: calcaneonavicular complex sagittal plane and rearfoot complex sagittal plane; calcaneocuboid transverse plane and rearfoot complex transverse plane; medial forefoot sagittal plane and calcaneonavicular complex frontal plane; and lateral forefoot sagittal plane and calcaneocuboid frontal plane (Table 1).

**Table 1:** Coupling angles

Couple		Stance Subphase Coupling Direction (°)			
**Distal joint complex** **Motion Plane**	**Proximal joint complex** **Motion Plane**	**Loading response**	**Midstance**	**Terminal stance**	**Pre-swing**

Calcaneonavicular Frontal	Rearfoot Frontal	149.45±30.98	234.34±50.18	232.74±72.60	243.19±134.42

Calcaneonavicular Transverse	Rearfoot Transverse	36.54±34.37	33.01±4.16	33.18±9.40	40.42±14.61

Calcaneocuboid Sagittal	Rearfoot Sagittal	149.67±44.46	214.90±107.08	225.71±108.97	211.40±41.84

Calcaneocuboid Frontal	Rearfoot Frontal	213.83±22.85	172.10±68.40	131.86±98.60	190.87±126.79

Calcaneocuboid Transverse	Rearfoot Transverse	287.89±98.31^a^	169.05±32.07^a^	150.01±19.73	160.40±45.48

Medial forefoot Sagittal	Calcaneonavicular Frontal	129.19±113.93	129.38±41.21^b^	182.49±43.12^bc^	256.82±19.86^c^

Lateral forefoot Sagittal	Calcaneocuboid Frontal	170.25±43.90	133.17±72.88	88.87±71.84^c^	230.62±90.23^c^

First MTP Sagittal	Medial forefoot Sagittal	262.83±77.59	184.36±125.82	129.68±83.16	112.24±7.60

^a^Significantly different loading response and midstance subphase coupling angles

^b^Signficantly different midstance and terminal stance subphase coupling angles

^c^Signficantly different terminal stance and pre-swing subphase coupling angles

## Conclusions

These results are clinically relevant due to the fact that a number of previous studies investigating joint coupling have only calculated a single coupling angle between the segments of interest. The single coupling angle has then been assumed to represent the coupling relationship throughout the stance phase. The results of the current study, however, suggest that this assumption may not be valid for all the coupling relationships between the distal foot segments during walking gait.
